# Factors influencing choice of treatment for proximal humeral fractures elaborated in a Delphi consensus process

**DOI:** 10.1007/s00402-023-05028-0

**Published:** 2023-09-02

**Authors:** Lisa Klute, Leopold Henssler, Benedikt Schliemann, Matthias Königshausen, Nadine Weber, Volker Alt, Maximilian Kerschbaum

**Affiliations:** 1 Department of Trauma Surgery, University Hospital Regensburg, Franz-Josef-Strauss-Allee 11, 93053 Regensburg, Germany; 2 Department of Trauma, Hand and Reconstructive Surgery, Herz-Jesu Hospital, 48165 Münster, Germany; 3 Department of Trauma Surgery and General Surgery, BG University Hospital Bergmannsheil, Ruhr University Bochum, Bürkle-de-La-Camp-Platz 1, 44789 Bochum, Germany

**Keywords:** Proximal humerus fracture, Delphi consensus, Shoulder surgery, Humeral head fractures

## Abstract

**Introduction:**

Hitherto, the decision-making process for treatment of proximal humerus fractures (PHF) remains controversial, with no established or commonly used treatment regimens. Identifying fracture- and patient-related factors that influence treatment decisions is crucial for the development of such treatment algorithms. The objective of this study was to define a Delphi consensus of clinically relevant fracture- and patient-related factors of PHF for clinical application and scientific research.

**Methods:**

An online survey was conducted among an international panel of preselected experienced shoulder surgeons. An evidence-based list of fracture-related and patient-related factors affecting treatment outcome after PHF was generated and reviewed by the members of the committee through online surveys. The proposed factors were revised for definitions, and suggestions from the first round mentioned in the free text were included as possible factors in the second round of surveys. Consensus was defined as having at least a two-thirds majority agreement.

**Results:**

The Delphi consensus panel consisted of 18 shoulder surgeons who completed 2 rounds of online surveys. There was an agreement of more than two-thirds of the panel for three fracture-related factors affecting treatment decision in the case of PHF: head-split fracture, dislocated tuberosities, and fracture dislocation. Of all patient-related factors, a two-thirds consensus was reached for two factors: age and rotator cuff tear arthropathy.

**Conclusion:**

This study successfully conducted a Delphi consensus on factors influencing decision-making in the treatment of proximal humeral fractures. The documented factors will be useful for clinical evaluation and scientific validation in future studies.

**Supplementary Information:**

The online version contains supplementary material available at 10.1007/s00402-023-05028-0.

## Introduction

Proximal humerus fractures are a common injury, particularly in the elderly, and can result in significant pain, functional impairment, and reduced quality of life [1, 2]. The management of proximal humerus fractures remains a subject of debate, with a range of available treatment options, including non-operative and operative approaches. Effective management of these fractures is crucial for optimal patient-reported outcome and quick return to functional activities. The treatment decision for proximal humerus fractures is influenced by various factors such as patient age, health status, type of fracture, and patient’s demands [3]. Currently, there is no established guideline for treatment decisions in PHF, so that choice of treatment for the individual patient remains challenging in clinical practice [4].

Non-operative treatment is commonly used in elderly patients or those with medical comorbidities that increase perioperative risk of surgical complications [5, 6]. Non-operative treatment usually includes sling immobilization of the affected arm for a period of time to allow initiation of fracture healing [7] and concomitant physical therapy to help maintain range of motion and prevent muscle wasting.

Surgical treatment of proximal humerus fractures is typically reserved for complex fractures, displaced fractures, or fractures with joint surface involvement [8, 9]. The goal of surgical treatment is to restore normal alignment and function of the shoulder, reduce pain, and prevent long-term complications such as stiffness, weakness, and osteoarthritis. The choice of surgical approach depends on the specific characteristics of the fracture, including the location, degree of displacement, number of fragments, and associated injuries [3, 10] and ultimately the preferences of the treating surgeon.

Recent literature suggests that an individualized and evidence-based approach is required for effective management of these fractures, taking relevant parameters into consideration such as the patient’s age, medical comorbidities, and functional goals [3, 4]. To investigate a current consensus among experts on the most important factors influencing the treatment decision of proximal humerus fractures, a Delphi consensus study was conducted. The Delphi consensus methodology is a well-established technique for achieving consensus among a panel of experts, and anonymous feedback and iterative rounds of questioning are used to refine and clarify opinions. This method has been successfully applied in various medical fields to reach agreement on best practices and guidelines.

## Methods

### Expert panel and mode of data collection

An expert panel of 18 orthopaedic shoulder and trauma surgeons from Austria, Germany, and Switzerland was convened to participate in the Delphi consensus process. The panel consisted of the members of the Research and Development Committee of the German, Austrian, and Swiss Shoulder and Elbow Association (DVSE). Each of the surgeons consulted had extensive experience in the treatment of PHF as well as exceptional research experience, making them highly qualified experts in the field. A steering committee was established with the two chairmen of the research and development committee of the DVSE and a statistician from our centre of clinical research to oversee and guide the Delphi process. A modified Delphi consensus procedure was used with two successive online surveys utilizing an online survey portal (SurveyMonkey, Momentive Inc., San Mateo, California, USA) for individual completion of the predefined questionnaire. For each round of the Delphi process, the panel received an online link to the questionnaire by email. The survey was conducted anonymously, with the exception that participants had knowledge of the included participants due to the invitation email, which addressed all members of the Research and Development Committee of DVSE. Taking this into account, the display of answers in the second round was anonymous [11].

### Literature review

A systematic literature review was conducted to identify the most relevant patient- and fracture-related factors influencing treatment decision of PHF. The systematic literature review was conducted by employing a comprehensive search strategy in PubMed and Medline databases to identify relevant studies on the factors influencing the treatment decision for proximal humerus fractures. The results are summarized in Table 1.

**Table 1:** Literature overview for treatment relevant factors in proximal humerus fractures

Factor	References
• 2-/3-/4-part fracture	Hertel et al. [12], Heers et al. [13], Hao et al. [10]
• Displacement of tuberosities	Hertel et al. [12], Resch et al. [14]
• Head-split fracture	Peters et al. [8], Hertel et al. [12], Resch et al. [14]
• Fracture-dislocation	Resch et al. [14], Hertel et al. [12]
• Varus/Valgus impaction	Resch et al. [14]
• Length of the medial metaphyseal head extension	Hertel et al. [12]
• Metaphyseal comminution zone	Hertel et al. [12], Jung et al. [15]
• Medial hinge dislocation	Hertel et al. [12]
• Fragment dislocation subacromial	McLaughlin et al. [16], Bono et al. [17]
• Acute lesion of rotator cuff	Schliemann et al. [18], Gallo et al. [19]
• Osteoporosis	Stolberg-Stolberg et al. [20], Carbone et al. [21]
• Age	Patel et al. [5], Spross et al. [3]
• Gender	Patel et al. [5], Walter et al. [22]
• High physical demand	Spross et al. [3]
• Multiple comorbidities	Garcia-Reza et al. [23]
• Dementia	Ballard et al. [24], Stolberg-Stolberg et al. [20]
• Dependency of nursing care	Hao et al. [10], Spross et al. [3]
• Physical disability of the contralateral arm or the legs	Pluijm et al. [25], Myeroff et al. [26]
• Palliative situation	Garrigues et al. [27]
• Substance addiction	Zhu et al. [28], Chakkalakal. et al. [29]
• Smoking	Porter et al. [30]
• Oral anticoagulation	Street et al. [31]

### First round of online survey

During the first round, the participants were invited to answer questions regarding their expertise in shoulder surgery such as surgical experience, treatment frequency of PHF, and whether they consider themselves as trauma surgeons or orthopaedic shoulder surgeons. After the self-assessment, evidence-based factors (see Table 1) were listed in two separate categories as fracture-related factors and patient-related factors. Participants were asked to select the five most relevant parameters from each list.

Additionally, panel members also commented on their opinion about relevant parameters to consider in the context of various treatment options and suggested adding further factors to the list that had not yet been included in the survey.

### Second round of online survey

In the beginning of the second round, the results of the initial round were displayed as a table with percentages and numbers of votes for every participant to review. Previously suggested factors from the free text fields were added to the list of selectable risk factors after consent had been obtained through a question beforehand. None of the possible answers from the first round were dismissed for the second round. Participants were then asked to select the most relevant factors again, considering the survey results from the first round and the added factors. To increase the possibility of a consensus, only three factors were allowed to be selected instead of five in the second round. There was again a possibility of inserting comments within free text fields.

### Data analysis and final adjudication

Data collection was performed using an online survey portal (SurveyMonkey, Momentive Inc., San Mateo, California, USA), and standard descriptive analyses of survey data were conducted using the SPPS software package version 25 (SPSS Inc, Chicago, Illinois). Consensus was operationally defined as the attainment of an agreement among a minimum of two-thirds of the participants. All comments and suggestions made by the participants were reviewed and evaluated by the study manager. The steering committee made final adjustments and corrections on the supervision protocol, considering improvements that increased accuracy, correctness and applicability.

## Results

**Fig. 1:**
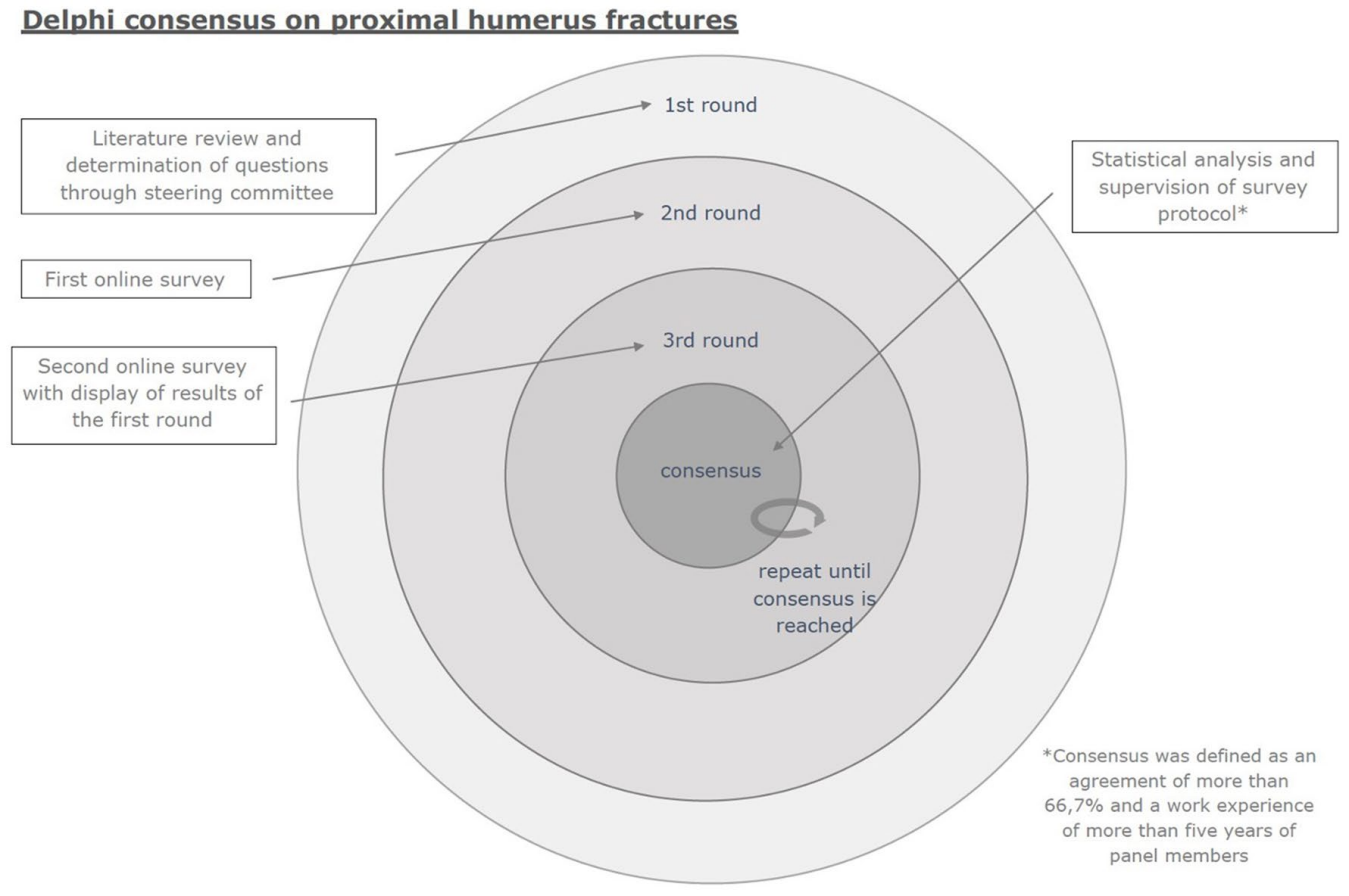
Methodological process of the conducted Delphi consensus

Eighteen members of the Research Committee of the German, Austrian and Swiss Shoulder and Elbow Society (DVSE) completed 100% of the questions in the two rounds of the Delphi consensus process. 50% of the participants (9/18) identified themselves as trauma surgeons and 50% as orthopaedic shoulder surgeons. While 61% (11/18) reported having work experience of 5–10 years, 33% (6/18) declared 10–20 years of experience and 6% (1/18) reported having experience of more than 20 years. The number of PHF treated per year (non-operative or surgical treatment) was ≤ 20 in 22% (4/18), 20–50 in 11% (2/18), 50–100 in 33% (6/18), and ≥ 100 in 33% (6/18). The process of the conducted Delphi method is shown in Fig. 1.

### First survey

The results of the first round of online survey are presented in Table 2.

**Table 2:** Fracture-related factors influencing treatment decisions of proximal humerus fractures and the frequency of their selection in the first online survey

Fracture-related factor	*n*	Percentage (%)
2-/3-/4-part fracture	7	38.89
Displacement of tuberosities	15	83.33
Head-split fracture	17	94.44
Fracture-dislocation	16	88.89
Varus/Valgus impaction	12	66.67
Length of the medial metaphyseal head extension	1	556
Metaphyseal comminution zone	9	50.00
Medial hinge dislocation	10	55.56
Fragment dislocation subacromial	2	11.11
Acute lesion of rotator cuff	0	0.00
Osteoporosis	1	5.56
Total	90	

After the first round, a consensus of more than two-thirds (see Table 2) was observed for displacement of the tuberosities (83.33%), head-split fracture (94.44%), fracture dislocation (88.89%) and varus/valgus impaction (66.67%).

Within the patient-related factors, a two-thirds consensus was achieved for age (83.33%) and physical disability of the contralateral arm or leg (66.67%) after the first round, as shown in Table 3.

**Table 3:** Patient-related factors influencing treatment decisions of proximal humerus fractures and the frequency of their selection in the first survey

Patient-related factor	*n*	Percentage (%)
Age	15	83.33
Gender	2	11.11
High physical demand	10	55.56
Multiple comorbidities	7	38.89
Dementia	9	50.00
Dependency of nursing care	7	38.89
Physical disability of the contralateral arm or the legs	12	66.67
Palliative situation	9	50.00
Substance addiction	7	38.89
Smoking	10	0.00
Oral anticoagulation	1	5.56
Total	90	

In the provided designated free text sections, the following parameters were stated as relevant by the respondents: “amount of cancellous bone in the calotte for anchoring screws”, “condition of the glenoid regarding associated fractures or defects”, and “the patient’s opinion”. One participant added that there were not enough possible answers to choose from in both categories. To complete the list of possible choices, a question in the second survey was added asking whether all participants would agree to add the suggested factors to this list.

### Second survey

The results of the second survey are presented in Table 4.

**Table 4:** Fracture-related factors influencing treatment decisions of proximal humerus fractures and the frequency of their selection in the second survey

Fracture-related factor	*N*	Percentage (%)
2-/3-/4-part fracture	0	0.00
Displacement of tuberosities	15	83.33
Head-split fracture	18	100
Fracture dislocation	17	94.44
Varus/Valgus impaction	2	11.11
Length of the medial metaphyseal head extension	0	0.00
Metaphyseal comminution zone	0	0.00
Medial hinge dislocation	1	5.56
Fragment dislocation subacromial	0	0.00
Acute lesion of rotator cuff	0	0.00
Osteoporosis	0	0.00
Condition of the calotte fragment for anchoring of screws	1	5.56
Condition of the glenoid	0	0.00
Total	90	

**Fig. 2:**
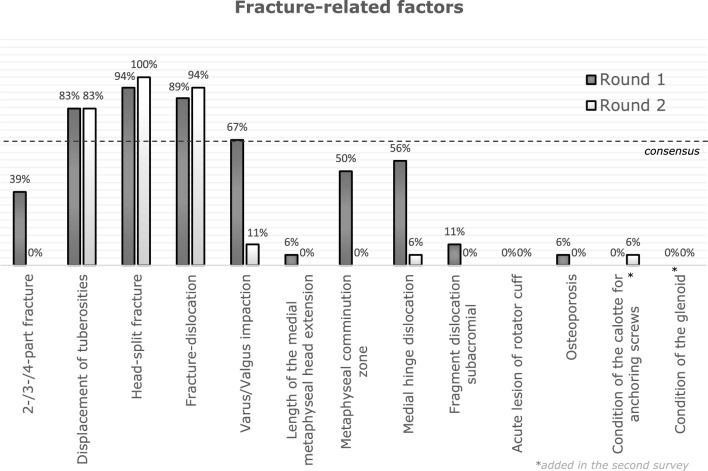
Results of the first and second survey for fracture-related factors

**Fig. 3:**
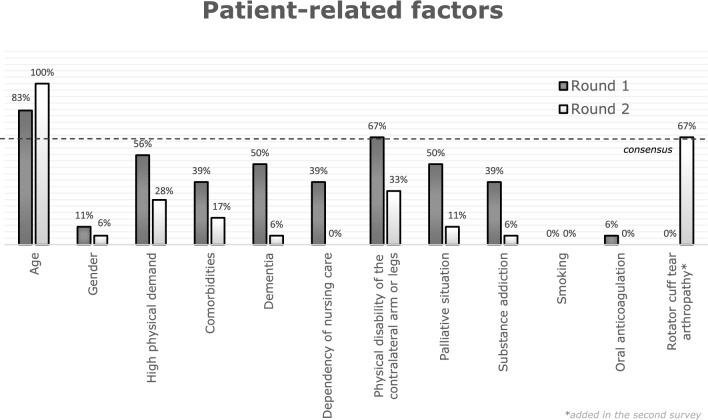
Results of the first and second survey for patient-related factors

A consensus of ≥ 66.7% was obtained for displacement of the tuberosities (83.33%), head-split fracture (100%) and fracture dislocation (94.44%) as shown in Table 4. In contrast to the first survey, “varus/valgus impaction” failed to reach a two-thirds consensus in the second round of survey. Moreover, the factors 2-/3-/4-part fracture, metaphyseal comminution zone and medial hinge dislocation were remarkably less frequently selected (Figs. 2 and 3).

Ultimately, after the second round, a consensus of at least two-thirds was achieved for age (100%) and rotator cuff tear arthropathy (66.67%), as shown in Table 5. Physical disability of the contralateral arm or legs as a patient-related factor did not reach the margin of consensus of 66.7% in the second survey, as it did before in the first round.

**Table 5:** Patient-related factors influencing treatment decisions of proximal humerus fractures and the frequency of their selection in the second survey

Patient-related factor	*n*	Percentage (%)
Age	18	100
Gender	1	5.56
High physical demand	5	27.78
Comorbidities	3	16.67
Dementia	1	5.56
Dependency of nursing care	0	0.00
Physical disability of the contralateral arm or legs	6	33.33
Palliative situation	2	11.11
Substance addiction	1	5.56
Smoking	0	0.00
Oral anticoagulation	0	0.00
Rotator cuff tear arthropathy	12	66.67
Patient’s opinion	5	27.78
Total	90	

In the designated free text section, “substance addiction” and “polytoxicomania” were mentioned as additional influencing factors. Since similar selection options had already been part of the list of patient-related factors, an addition of the suggested factors was not considered appropriate.

## Discussion

Consensus in this Delphi process for treatment of PHF was obtained for fracture-related factors, including head-split fracture, dislocated tuberosity, and fracture dislocation, as well as patient-related factors, such as age and rotator cuff tear arthropathy.

Proximal humerus fractures are a common injury with an incidence of 6.6 cases per 1000 person years. Thus, PHF are the third most common fracture type in patients over 60 years of age, what accounts for 4–6% of all fractures [1].

While there is still controversy regarding the right treatment for PHF [2], a structural evaluation of relevant treatment influencing factors could assist in decision-making for clinicians who are performing therapy in patients with PHF.

Head-split fractures refer to fractures that involve at least 20% of the articular surface of the humeral head and extend into the metaphysis [12]. These fractures are often challenging to manage, as they can involve the blood supply to the humeral head and are associated with elevated rates of avascular necrosis [12]. The Delphi consensus study found that the head-split component of humeral head fractures was important to consider in the decision-making process for selecting the treatment of proximal humerus fractures. For patients with head-split fractures, surgical intervention may be recommended [8], as non-surgical management is associated with a higher risk of complications, including avascular necrosis [12]. However, the choice of surgical intervention may depend on the extent of the fracture, the patient’s age, and their functional demands [2, 7].

The tuberosities are bony protrusions on the proximal end of the humerus, which provide attachment sites for the rotator cuff tendons. Dislocation of any one of the tuberosities can significantly impact the management of the fracture [32, 33]. For patients with a dislocated tuberosity, surgical intervention may be recommended to restore the attachment of the rotator cuff tendons [34]. However, this may depend on the extent of the fracture and the patient’s age and condition of the rotator cuff. A recently published randomized controlled trial with long-term outcome evaluation of dislocated PHF with affected tuberosities showed that there was no better outcome in patients who were surgically treated [2]. Nevertheless, there are other studies that indicate better functional outcome after surgical treatment for those PHF [9]. Regarding reverse shoulder arthroplasty for treatment of PHF, dislocated tuberosities can have a high impact on the surgical results [32].

A fracture dislocation of the proximal humerus refers to a severe injury where the humeral head is dislocated from its socket in the glenoid and also sustains a fracture. These fractures can be challenging to manage, as they involve both the proximal humerus and the shoulder joint [35]. The current Delphi consensus study found that an additional occurrence of glenohumeral dislocation is also considered a clinically important factor in the decision-making process for treatment of proximal humerus fractures. Fracture dislocations of the shoulder are often treated surgically because they can be associated with significant displacement and comminution of the fragments [35], as well as damage to the surrounding soft tissue structures, such as the rotator cuff [36]. Yet, the kind of treatment varies depending on the patient’s age, bone density and associated bony defects [35, 37].

Patient age, as a patient-related factor which achieved consensus in our conducted Delphi process, is known to be an important factor that influences the management of proximal humerus fractures [5]. Younger patients tend to have better bone quality and may be able to tolerate surgical intervention better, while older patients may have poorer bone quality [6] and may be at a higher risk of perioperative and postoperative complications such as need for revision and non-union [38]. For younger patients, surgical intervention may be recommended and head-preserving treatment should be the prioritized aim for this patient group. However, in older patients, non-operative management may be considered in most cases apart from fracture dislocations, especially if patients have pre-existing comorbidities [2].

Rotator cuff tear arthropathy refers to the development of osteoarthritis as a result of massive rotator cuff tears and subsequent cranialization of the humeral head. The existence of rotator cuff tear arthropathy can significantly impact the treatment decisions in the management of proximal humerus fractures, as it can limit the applicability of head-preserving treatment options due to poor expectable functional results and may require more elaborate treatment. For patients with significant rotator cuff tear arthropathy, shoulder replacement surgery should be considered to address the arthritic changes and the loss of function of the rotator cuff [39]. However, careful consideration is needed when selecting patients for this shoulder replacement surgery [40].

This survey is constrained by the limitations of the Delphi method in general, including the selection of the panel, the definitions of the factors and questions. Also, the conduct of the survey, the analysis of the responses, and the final decision are bound to the Delphi method.

At the same time, conducting the survey within the Research and Development Committee of the German, Austrian and Swiss Shoulder and Elbow Society (DVSE) with a 100% response rate can be considered a major strength of this investigation, as a high level of experience among the participants could be assumed. While there is a prior Delphi study focussing on exploring post-treatment complications associated with proximal humerus fractures [41], we are not aware of any similar work that has been conducted to evaluate treatment influencing factors in PHF of shoulder experts. Hao et al. evaluated in 2021 how differently orthopaedic shoulder and trauma surgeons decide in their treatment options for PHF and what factors they found relevant [10]. They reported that non-surgical management or reverse shoulder arthroplasty were the preferred treatment regimens for elderly patients with complex fracture patterns and poor bone quality, osteoarthritis, or rotator cuff dysfunction [10]. Fractures with good bone quality of younger patients were preferentially treated with osteosynthesis or hemiarthroplasty [10]. Cosic et al. recently showed better quality of life for surgically treated patients with highly displaced PHF [9]. This disagrees with the results of the ProFHER study, in which surgical therapy for PHF was not able to show superiority in the long term [2].

In Switzerland, Spross et al. conducted a prospective study in 2019 of the treatment of PHF based on a treatment algorithm they developed on their own [3]. In the first step of their algorithm, they distinguish between young healthy and active patients, usually under 65 years of age, and older patients over 65 years of age. This emphasizes the relevance of this factor. In young patients, the method of treatment is then selected based on the fracture pattern. For older patients, further patient-related factors are relevant in the decision-making algorithm [3].

It is important to state that the management of proximal humerus fractures should be individualized based on the patient’s unique circumstances. A treatment algorithm solely based on a fracture classification therefore is not appropriate and is not alone sufficient for clinical application. Factors such as the patient’s overall health status, functional demands, and expectations for recovery should also be considered in the decision-making process. Shared decision-making between the patient and their healthcare provider can help ensure that the treatment plan aligns with the patient’s goals and demands.

## Conclusion

In conclusion, this Delphi consensus study provides valuable insights into the factors that influence the decision-making process for proximal humerus fractures in clinical daily practice of experienced shoulder surgeons. Age, rotator cuff tear arthropathy, head-split fractures, dislocated tuberosities, and fracture-dislocations were identified as important factors that are considered when deciding on the optimal management strategy for these fractures. While these factors provide a helpful framework for decision-making, it is important to consider each patient’s unique circumstances and preferences when developing a treatment plan. Further investigation is needed to validate the importance of these factors.

### Supplementary Information

Below is the link to the electronic supplementary material.

Supplementary file1 (PDF 38 KB)

Supplementary file2 (PDF 156 KB)

## Data Availability

The data supporting this study are available upon request.
