# Supplementary material for: Factors influencing choice of treatment for proximal humeral fractures elaborated in a Delphi consensus process
Source: Arch Orthop Trauma Surg. 2023 Sep 2;143(12):7053–61. doi: 10.1007/s00402-023-05028-0 (PMC10635939; doi:10.1007/s00402-023-05028-0)
Supplement: Supplementary file 1 — Supplementary file1 (PDF 38 KB) [file 402_2023_5028_MOESM1_ESM.pdf]

Delphi-Konsens zu proximalen Humerusfrakturen

* 1. Willigen Sie der Teilnahme an diesem Delphi-Prozess zu proximalen Humerusfrakturen ein?

◯ Ja

◯ Nein

* 2. Wie viele Jahre Berufserfahrung in der Unfallchirurgie/Schulterchirurgie haben Sie?

◯ maximal 5 Jahre

◯ 5-10 Jahre

◯ 10-20 Jahre

◯ mindestens 20 Jahre

* 3. Wie viele proximale Humerusfrakturen behandeln Sie in etwa pro Jahr (konservativ und operativ)?

◯ maximal 20

◯ 20-50

◯ 50-100

◯ mindestens 100

* 4. Bezeichnen Sie sich als orthopädische/r Schulterchirurgin/Schulterchirurg oder Unfallchirurgin/Unfallchirurg?

◯ Orthopäde/-in

◯ Unfallchirurg/in

* 5. Bitte versuchen Sie die **5 wichtigsten von den unten genannten Frakturkriterien** zu identifizieren und als relevant zu benennen.

Das Ziel ist hierbei die Evaluierung der Frage, wie wichtig die einzelnen Faktoren für Sie zur Indikationsstellung bei proximalen Humerusfrakturen sind, um zwischen den verschiedenen Behandlungsmethoden (konservativ, osteosynthetisch, endoprothetisch) zu entscheiden.

- [ ] 2-/3-/4-Part-Fraktur [1]
- [ ] Dislozierte Tuberkula [2]
- [ ] Headsplit-Fraktur [3]
- [ ] Luxationsfraktur [2]
- [ ] Varische / valgische Stellung [4]
- [ ] Posteromediale Extension [5]
- [ ] Metaphysäre Trümmerzone [5]
- [ ] Zerstörung des medialen Scharniers [5]
- [ ] Fragmentdislokation subakromial
- [ ] Akute Läsion der Rotatorenmanschette
- [ ] Osteoporose

Anmerkungen und Ergänzungen

* 6. Bitte versuchen Sie die **5 wichtigsten patientenassoziierten Faktoren** aus den unten genannten Kriterien zu identifizieren und als relevant zu benennen.

Das Ziel ist hierbei die Evaluierung der Frage, wie wichtig die einzelnen Faktoren für Sie zur Indikationsstellung bei proximalen Humerusfrakturen sind, um zwischen den verschiedenen Behandlungsmethoden (konservativ, osteosynthetisch, endoprothetisch) zu entscheiden.

- [ ] Alter
- [ ] Geschlecht
- [ ] Hoher körperlicher Anspruch
- [ ] Multiple Vorerkrankungen
- [ ] Demenz Pflegebedürftigkeit
- [ ]
- [ ] Körperliche Behinderung des kontralateralen Armes oder der Beine
- [ ] Palliative Situation
- [ ] Substanzabhängigkeit
- [ ] Nikotinabusus
- [ ] Orale Antikoagulation
- [ ] Defektarthropathie

Anmerkungen und Ergänzungen

7. Gibt es Ihrer Meinung nach weitere Faktoren, welche unbedingt die Therapieentscheidung beeinflussen? Wenn ja, bitten wir Sie, diese hier zu ergänzen.

8. Haben Sie Anmerkungen zu den oben erwähnten therapierelevanten Faktoren oder Ergänzungen?

Erläuterungen

[1] 2-/3-/4-Part-Frakturen nach Codman von 1934 mit Bezeichnung der 4 relevanten Hauptfragmente a: Tuberculum majus, b: Tuberculum minus, c: Kalotte, d: Schaft.

{Codman EA (1934) The Shoulder: Rupture of the supraspinatus tendon and other lesions in or about the subacromial bursa. T. Todd Company, Boston}.

[2] Neer-Klassifikation der proximalen Humerusfrakturen mit Einteilung derjenigen Frakturen mit Dislokation der Tuberkula sowie Luxationsfrakturen.

{Habermeyer P (1997) Die Humeruskopffraktur. Unfallchirurg 100:820–837}.

[3] Headsplit-Fraktur als mehrfragmentäre Frakturentität des Kalottenfragments; Headsplit-Klassifikation nach Scheibel.

{Scheibel, M., Peters, P., Moro, F. et al. Head-split fractures of the proximal humerus. Obere Extremität 14, 93–102 (2019). https://doi.org/10.1007/s11678-019-0520-6}.

[4] Varische oder valgische Fehlstellung in der coronaren Ebene der radiographischen Bildgebung, zum Beispiel erhoben anhand des Head-Shaft-Angles (Kopf-Schaft-Winkel im Bild entsprechend CCD) nach Hertel.

{Hertel R, Knothe U, Ballmer FT. Geometry of the proximal humerus and implications for prosthetic design. J Shoulder Elbow Surg. 2002 Jul-Aug;11(4):331-8. doi: 10.1067/mse.2002.124429. PMID: 12195250.}.

[5] Hertel-Kriterien zur Evaluation des Risikos einer Humeruskopfnekrose nach proximaler Humerusfraktur mit a: posteromedialer Extension unter oder über 8 mm, b: Zerstörung des medialen Scharniers („medial Hinge-Dislokation“), c: Vorhandensein einer metaphysären Trümmerzone.

{Hertel R, Hempfing A, Stiehler M, Leunig M. Predictors of humeral head ischemia after intracapsular fracture of the proximal humerus. J Shoulder Elbow Surg. 2004 Jul-Aug;13(4):427-33. doi: 10.1016/j.jse.2004.01.034. PMID: 15220884.}
