# Supplementary material for: Factors influencing choice of treatment for proximal humeral fractures elaborated in a Delphi consensus process
Source: Arch Orthop Trauma Surg. 2023 Sep 2;143(12):7053–61. doi: 10.1007/s00402-023-05028-0 (PMC10635939; doi:10.1007/s00402-023-05028-0)
Supplement: Supplementary file 2 — Supplementary file2 (PDF 156 KB) [file 402_2023_5028_MOESM2_ESM.pdf]

Runde 2 des Delphi-Konsens zu proximalen Humerusfrakturen

Sehr geehrte Kolleginnen und Kollegen,
vielen Dank für Ihre Teilnahme an der ersten Runde dieses Delphi-Konsens-Prozesses zur Behandlung von proximalen Humerusfrakturen.

Im Nachfolgenden werden Ihnen die Ergebnisse der 18 Teilnehmenden der ersten Umfragerunde präsentiert.

Runde 2 des Delphi-Konsens zu proximalen Humerusfrakturen

Ergebnisse der ersten Runde für die frakturassoziierten Faktoren

ANTWORTOPTIONEN	BEANTWORTUNGEN	
2-/3-/4-Part-Fraktur [1]	38.89%	7
Dislozierte Tuberkula [2]	83.33%	15
Headsplit-Fraktur [3]	94.44%	17
Luxationsfraktur [2]	88.89%	16
Varische / valgische Stellung [4]	66.67%	12
Posteromediale Extension [5]	5.56%	1
Metaphysäre Trümmerzone [5]	50.00%	9
Zerstörung des medialen Scharniers [5]	55.56%	10
Fragmentdislokation subakromial	11.11%	2
Akute Läsion der Rotatorenmanschette	0.00%	0
Osteoporose	5.56%	1
Befragte insgesamt: 18		

Somit wurde ein Konsens für die folgenden drei Faktoren erlangt:

- Headsplit-Fraktur
- Luxationsfraktur
- Dislozierte Tuberkula

Runde 2 des Delphi-Konsens zu proximalen Humerusfrakturen

In den Freitextfeldern wurden folgende Faktoren ergänzt:
- Kalottenbeschaffenheit zur Verankerungsmöglichkeit
- Glenoidbeschaffenheit

Diese werden nun in die Liste der potentiell relevanten frakturassoziierten Faktoren aufgenommen.
Trotz des bereits erlangten Konsens für die genannten drei Faktoren muss nun nach Ergänzung der neuen Faktoren erneut der Konsens abgefragt werden.

Runde 2 des Delphi-Konsens zu proximalen Humerusfrakturen

* 1. Bitte versuchen Sie nun vor dem Hintergrund der Umfrageergebnisse der ersten Runde erneut die für Sie **3 wichtigsten Frakturkriterien** zu identifizieren und als relevant zu benennen.
Die Prozentangaben in Klammern stellen die Umfrageergebnisse der letzten Runde dar.

Das Ziel ist hierbei die Evaluierung der Frage, wie wichtig die einzelnen Faktoren für Sie zur Indikationsstellung bei proximalen Humerusfrakturen sind, um zwischen den verschiedenen Behandlungsmethoden (konservativ, osteosynthetisch, endoprothetisch) zu entscheiden.

- ☐ 2-/3-/4-Part-Fraktur (39%)
- ☐ Dislozierte Tuberkula (83%)
- ☐ Headsplit-Fraktur (94%)
- ☐ Luxationsfraktur (89%)
- ☐ Varische / valgische Stellung (67%)
- ☐ Posteromediale Extension (6%)
- ☐ Metaphysäre Trümmerzone (50%)
- ☐ Zerstörung des medialen Scharniers (56%)
- ☐ Fragmentdislokation subakromial (11%)
- ☐ Akute Läsion der Rotatorenmanschette (0%)
- ☐ Osteoporose (6%)
- ☐ Kalottenbeschaffenheit zur Verankerungsmöglichkeit
- ☐ Glenoidbeschaffenheit

Anmerkungen und Ergänzungen

☐

Runde 2 des Delphi-Konsens zu proximalen Humerusfrakturen

Ergebnisse der ersten Runde für die patientenassoziierten Faktoren

ANTWORTOPTIONEN	BEANTWORTUNGEN	
Alter	83.33%	15
Geschlecht	11.11%	2
Hoher körperlicher Anspruch	55.56%	10
Multiple Vorerkrankungen	38.89%	7
Demenz	50.00%	9
Pflegebedürftigkeit	38.89%	7
Körperliche Behinderung des kontralateralen Armes oder der Beine	66.67%	12
Palliative Situation	50.00%	9
Substanzabhängigkeit	38.89%	7
Nikotinabusus	0.00%	0
Orale Antikoagulation	5.56%	1
Defektarthropathie	61.11%	11
Befragte insgesamt: 18		

Somit wurde ein Konsens für den folgenden Faktor erlangt:

- Alter

Runde 2 des Delphi-Konsens zu proximalen Humerusfrakturen

In den Freitextfeldern wurde folgender Faktor ergänzt:
- Patientenwunsch

Dieser wird nun in die Liste der potentiell relevanten patientenassoziierten Faktoren aufgenommen.
Trotz des bereits erlangten Konsens für den genannten Faktor muss nun nach Ergänzung des neuen Faktors erneut der Konsens abgefragt werden.

Runde 2 des Delphi-Konsens zu proximalen Humerusfrakturen

* 2. Bitte versuchen Sie nun vor dem Hintergrund der Umfrageergebnisse der ersten Runde erneut die für Sie **3 wichtigsten patientenassoziierten Faktoren** aus den unten genannten Kriterien zu identifizieren und als relevant zu benennen.
Die Prozentangaben in Klammern stellen die Umfrageergebnisse der letzten Runde dar.

Das Ziel ist hierbei die Evaluierung der Frage, wie wichtig die einzelnen Faktoren für Sie zur Indikationsstellung bei proximalen Humerusfrakturen sind, um zwischen den verschiedenen Behandlungsmethoden (konservativ, osteosynthetisch, endoprothetisch) zu entscheiden.

- ☐ Alter (83%)
- ☐ Geschlecht (11%)
- ☐ Hoher körperlicher Anspruch (56%)
- ☐ Multiple Vorerkrankungen (39%)
- ☐ Demenz (50%)
- ☐ Pflegebedürftigkeit (39%)
- ☐ Körperliche Behinderung des kontralateralen Armes oder der Beine (67%)
- ☐ Palliative Situation (50%)
- ☐ Substanzabhängigkeit (39%)
- ☐ Nikotinabusus (0%)
- ☐ Orale Antikoagulation (6%)
- ☐ Defektarthropathie (61%)
- ☐ Patientenwunsch

Anmerkungen und Ergänzungen

☐

Runde 2 des Delphi-Konsens zu proximalen Humerusfrakturen

3. Gibt es Ihrer Meinung nach weitere Faktoren, welche unbedingt die Therapieentscheidung beeinflussen? Wenn ja, bitten wir Sie, diese hier zu ergänzen.

4. Haben Sie Anmerkungen zu den oben erwähnten therapierelevanten Faktoren oder Ergänzungen?
